# Prevalence and pattern of refractive errors among Saudi adults

**DOI:** 10.12669/pjms.35.2.648

**Published:** 2019

**Authors:** Mujeeb Ur Rehman Parrey, Ekramy Elmorsy

**Affiliations:** 1 *Dr. Mujeeb Ur Rehman Parrey, Ph.D. Department of Surgery, P.O. Box-1321, Faculty of Medicine, Northern Border University, Arar, Kingdom of Saudi Arabia*; 2 *Dr. Ekramy Elmorsy, MD. Department of Pathology, P.O. Box-1321, Faculty of Medicine, Northern Border University, Arar, Kingdom of Saudi Arabia*

**Keywords:** Ametropia, Astigmatism, Emmetropia, Hyperopia, Myopia, Refractive error, Visual impairment, **RE:** Refractive Error, **SE:** Spherical Equivalent, **VA:** Visual Acuity, **VI:** Visual Impairment

## Abstract

**Background & Objectives:**

Refractive Errors (RE) are responsible for major portion of the treatable visual impairment and avoidable blindness in the world. The prevalence of RE varies with age, gender, ethnicity, geographical locations and also from time to time due to progresse in eye care services. We aimed to study the prevalence of RE and assess their patterns among Saudi adults of Arar city, the capital of Northern Border Region of Saudi Arabia.

**Methods:**

This is a cross-sectional, population-based study. A total number of 966 Saudi adults aged 16 to 39 years were enrolled. The patterns of their RE were studied through auto-refraction evaluation.

**Results:**

The prevalence of RE was 45.8%. The most frequent type of RE was myopia in 24.4%, followed by hyperopia 11.9% and astigmatism in 9.5% cases. Ages and genders significantly affect the prevalence of the different patterns of RE (0.033 and 0.012, respectively).

**Conclusion:**

The prevalence of RE in Arar city is slightly lower than that previously published in the same targeted age group. Myopia is the main RE. More awareness programs, especially among young adults are recommended for better outcomes.

## INTRODUCTION

When the parallel rays of light coming from infinity are focused on the retina with the accommodation of the eye at rest it indicates the normal refractive status of the eye called as emmetropia. An emmetropic eye will therefore make a clear image of the distant object without any internal adjustment of its optics. If the rays of light with accommodation of the eye at rest are not focused exactly on the retina the condition indicates an error of refraction, which is referred to as ametropia.^1^ Refractive error (RE) is classified into myopia, hyperopia and astigmatism. In myopia with accommodation relaxed, light rays from an object at infinity are focused in front of retina and in hyperopia behind the retina while as in astigmatism the light rays do not focus at a single point because of variations in the curvature of the cornea or lens at different meridians.^2^

RE can be treated by optical methods like corrective glasses and contact lenses or surgical methods like LASIK (laser-assisted in situ keratomileusis) or PRK (photorefractive keratectomy).

Lack of knowledge and awareness about RE^3^, non-recognition of the problem at personal or family level, as well as at community and public health level; economic and social barriers and availability and affordability of eye health services are the main reasons for RE to remain uncorrected.^4^,^5^ RE have serious impact on economy of many countries of the world.^6^,^7^ Many studies conducted abroad indicate that the prevalence of RE exhibits significant variation across geographic, racial, age, gender and ethnic boundaries, which has an enormous impact on the strategies utilized in addressing the problem of RE.^8^,^9^ This is more likely in people living in countries with limited resources and poor access to the eye care services. Studies conducted in some parts of Saudi Arabia indicate that RE are among the leading causes of VI.^10^-^12^ In Saudi Arabia, RE were studied considering either mainly the pediatric population^13^-^16^ or particular population groups like students undergoing higher education.^17^

Although a large number of studies pertaining to RE have been conducted in many parts of the world, the comparison of the data remains difficult owing to lack of consistency in methods and definitions used for identifying and measuring RE. However, prevalence and patterns of RE in Arar city have not been studied yet. The data from the current study shall fill the gap to evaluate the current state of the problem in Arar city with better guidelines for the future strategies to overcome the burden of RE.

## METHODS

This cross-sectional, population-based study was conducted from January 1^st^, 2018 to September 1^st^, 2018. A sample from the general population was randomly collected at screening camps held in the main shopping mall of Arar city where a temporary eye clinic was installed for three consecutive days. Persons with any previous history of refractive surgery were excluded from this study. The participants were further evaluated at the Central Hospital of Arar city. Visual Acuity (VA) was tested on VA Auto Chart Projector (TOPCON ACP-8; Japan) and refraction without cyloplegia was performed on auto-refractor (Topcon KR-8900; Japan).

The RE was classified using the Spherical equivalent (SE), which is the sum of the value of the sphere and half of the cylindrical value.^18^ Emmetropia was attributed to SE between -0.50 D and +0.50 D, myopia to SE ≤ -0.50 D, hyperopia to SE ≥ +0.50D and astigmatism to any cylindrical error of at least 0.5 D without reference to the axis. Myopia was further categorized as low (≥ −0.50 D and < −3.00 D), moderate (≥ −3.00 D and < −6.00 D) and high (≥ −6.00 D). Hyperopia was further categorized as low to moderate (≥ +0.50 D and < +3.00 D) and high (≥ +3.0 D) hyperopia. Astigmatism was further categorized as low to moderate (cylinder error of ≥ 0.50 D and < 3.00 D) and high (≥ 3.00 D) astigmatism. Simple myopic astigmatism was defined as plano sphere (<−0.5 D to < +0.5 D) and cylinder of ≥ −0.50 D, simple hyperopic astigmatism was defined as plano sphere (< −0.5 D to < +0.5 D) and cylinder of ≥ +0.50 D); compound myopic astigmatism was defined as sphere of ≥ −0.5 D and cylinder of ≥ −0.50 D, compound hyperopic astigmatism was defined as sphere of ≥ +0.5 D and cylinder of ≥ +0.50 D. Astigmatism was defined as mixed if the sphere was positive (> +0.5 D) and cylinder value was negative (> −0.50 D) or vice versa and the cylinder value was greater than a sphere.

### Data Analysis

Data was revised, coded, entered, tabulated, and analyzed using SPSS version 20. Chisquare was used to study the significance of association. Statistical significance was kept constant at P<0.05.

This study was approved by the ethical committee of Deanship of Scientific Research, Northern Border University. Informed written consent was obtained from all participants involved in this study. Financial or any other compensations were not offered to any of the participants.

## RESULTS

**Fig.1:**
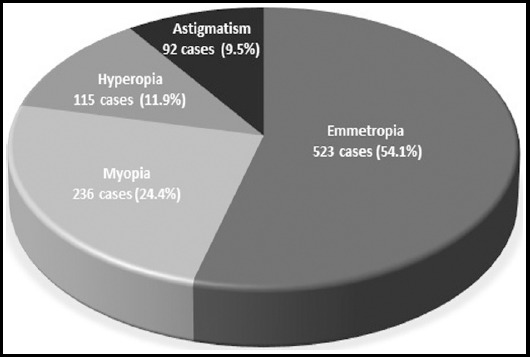
Refractive error among the studied population. Emmetropia was attributed to SE between -0.50D and +0.50D, myopia to SE ≤ -0.50D, hyperopia to SE ≥ +0.50D and astigmatism to any cylindrical error of ≤ -0.50D or ≥ +0.50D with SE in the emmetropia range without reference to the axis.

A total of 966 Saudi adults were enrolled in the current study [485 females (48.7%) and 481 males (51.3%)]. The mean age of the studied population was 27.48 (± 6.32) years with a range of 16-39 years old. The prevalence of RE was estimated to be 45.8%. The commonest type of RE was myopia (SE ≤-0.5D) in 24.4% followed by hyperopia (SE≥0.5D) in 11.9% and simple astigmatism (with SE between 0.5D and -0.5D) in 9.5% cases (Figure 1).

Regarding the genders of participants, there was a significant difference between the males and females in prevalence of RE (Table-I). Pertaining to the age (Table-II), myopia was found to be slightly more common in age group ≥ 25, while as hyperopia in >25 (p= 0.033). Grading of RE severity in the studied population is shown in Table-III. Cases with astigmatism were classified according to their SE (Table-I and II) and the commonest type was found to be the compound myopic astigmatism.

**Table-I:** Pattern of refractive error in relation to gender.

Type of refractive error	Total	Females	Males	P-value
Emmetropia	523 (54.1%)	240 (24.8%)	283 (29.3%)	0.0123* 17.93, 7
Myopia	67 (6.9)	42 (4.3%)	25 (2.6%)	
Hyperopia	31 (3.2%)	16 (1.7%)	15 (1.6%)	
Simple myopic astigmatism	76 (7.9%)	32 (3.3%)	44 (4.6%)	
Simple hyperopic astigmatism	16 (1.7%)	9 (0.9%)	7 (0.7%)	
Compound myopic astigmatism	166 (17.2%)	101 (10.5%)	65 (6.7%)	
Compound hyperopic astigmatism	8 (0.8%)	4 (0.4%)	4 (0.4%)	
Mixed astigmatism	79 (8.2%)	41 (4.2%)	38 (3.9%)	

Totals	966 (100%)	485 (50.2%)	481 (49.8%)	

**Table-II:** Pattern of refractive errors in relation to age groups.

Type of refractive error	Total	≥25 yrs.	>25 yrs.	P-value
Emmetropia	523 (54.1%)	293 (30.3%)	228 (23.6%)	0.033* 15.17 , 7
Myopia	67 (6.9)	37 (3.8%)	30 (3.1%)	
Hyperopia	31 (3.2%)	11 (1.1%)	20 (2%)	
Simple myopic astigmatism	76 (7.9%)	34 (3.5%)	32 (3.3%)	
Simple hyperopic astigmatism	16 (1.7%)	7 (0.7%)	9 (0.9%)	
Compound myopic astigmatism	166 (17.2%)	76 (7.9%)	90 (9.3%)	
Compound hyperopic astigmatism	8 (0.8%)	3 (0.3%)	5 (0.5%)	
Mixed astigmatism	79 (8.2%)	33 (3.4%)	46 (4.8%)	

Totals	966 (100%)	485 (50.2%)	481 (49.8%)	

**Table-III:** Severity of refractive errors in relation to gender and age groups.

RE	Severity	Totals	Females	Males	P-value	≥25 yrs.	>25 yrs.	P-value
Myopia	Mild	162(68.5%)	132(55.9%)	30(12.7%)	<0.0001	77 (32.6%)	85(35.9%)	0.0003
	Moderate	60 (25.4%)	15 (6.3%)	45 (19.1%)		46 (19.5%)	14(5.9%)	
	Severe	16 (6.7%)	12 (5%)	4 (1.7%)		11 (4.6%)	5 (2.1%)	
	Totals	236 (100%)	159(64.4%)	77 (35.6%)		134(56.8%)	102(43.2%)	
Hyperopia	Low to moderate	93 (79.9%)	48 (41.3%)	45 (38.6%)	1	31 (29.6%)	62 (50.3%)	1
	High	22 (19.1%)	11 (9.5%)	11(9.5%)		7 (6.1%)	15 (13%)	
	Totals	115 (100%)	59 (51.3%)	56 (48.7%)		38 (33%)	77(67%)	
Astigmatism	Low to moderate	324 (94%)	147(42.6%)	177(57.4%)	0.652	175(50.7%)	139(49.3%)	0.068
	High	21 (6%)	11 (3.2%)	10 (2.8%)		7 (2%)	14 (4%)	
	Totals	345 (100%)	158(45.8%)	187(54.2%)		182(52.8%)	153(47.2%)	

Cases with SE≤-0.5D were classified according to the cylindrical correction to simple myopia (cylinder>-0.5D and <0.5D), compound myopic stigmatism (cylindrical correction≤-0.5D) and mixed myopic stigmatism (cylindrical correction≥0.5D) (Table-IV).Cases with SE≥0.5D were classified according to the cylindrical correction to simple hyperopia (cylinder>-0.5D and <0.5D), compound hyperopic stigmatism (cylinder≥0.5D) and mixed hyperopic stigmatism (cylinder≤-0.5D) (Table-V).

**Table-IV:** Classification of cases with SE≤-0.5D among the studied population.

SE and cylindrical lenses	Number of cases
Simple myopia (cylinder>-0.5D and <0.5D)	67 (28.4%)
Compound myopic stigmatism (cylinder≤-0.5D)	166 (70.3%)
Mixed myopic stigmatism (cylinder≥0.5D)	3 (1.3%)

Total	236 (100%)

**Table-V:** Classification of cases with SE≥0.5D among the studied population.

SE and cylindrical lenses	Number of cases
Simple hyperopia (cylinder>-0.5D and <0.5D)	31 (26.9%)
Compound hyperopic stigmatism (cylinder≥0.5D)	8 (6.9%)
Mixed hyperopic stigmatism (cylinder≤-0.5D)	76 (66%)

Total	115 (100%)

## DISCUSSION

This study has evaluated the patterns and prevalence of RE in Arar city. The prevalence of RE was estimated to be 45.8%. The commonest type of RE was myopia (SE ≤-0.5D) in 24.4% followed by hyperopia (SE≥0.5D) in 11.9% and simple astigmatism (with SE between 0.5D and -0.5D) in 9.5% cases. Both genders and age groups of participants showed significant effect on patterns and prevalence of the different RE types.

No definite published data regarding the prevalence of RE is avaliable in Saudi Arabia. The published data were based only on school children and adolescents.^19^ The prevalence of RE in Saudi adults of Arar city is 45.8% which is lower than the prevalence of RE (72.2%) among female students of Medicine and Pharmacy Schools in Quassin University as reported by Albatanony.^20^ Another study from Quassim estimated the prevalence of RE as 58.6% among the male medical students of Qassim University.^17^ This higher prevalnce may be due to faulty abuse of vision during prolonged hours of studying among the medical students Kumar et al.,^21^ and Basu et al., 22 In an another study conducted in Riyadh^19^, the prevealence of RE among adolescents (12-20 years) was estimated to be 55.5% which is also higher than the present prevelence. In the nearby country Jordan, the prevalence of RE among adult population aged (17-40 years) was estimated to be around 60% (Mallen et al, 2005).^23^ Internationally, the prevalence in USA was reported to be around 72% in civilain population aged above 12 years (Vitale et al., 2008).^24^ This higher prevalence in USA may be due to the elderly age group (above 40 years) in their study.

In the current study myopia was the commonest type of RE. This is in accordance with some recent studies conducted in KSA^17^,^19^ and abroad.^25^,^26^ In Europe the greatest burden of RE is due to myopia.^27^ While other data showed higher prevalence of astigmatism.^28^,^29^ Higher prevalence of hyperopia is usually seen in studies targeting elder age groups than our study age range.^27^,^30^ However, all these differences regarding the prevalence of the different patterns of RE may be related to the difference in the studied populations, methods of study and ages of the studied groups.

As regards the effect of age on the patterns of RE, our data showed that myopia was more prevalent among participants aged <25 years, while hyperopia was more dominant in the elderly participants. This is in line with the previously published data.^17^,^25^-^27^,^30^

Regarding gender the current results had found that myopia was more common in females. This is in accordance with the other studies as Katz et al. (1997)^31^ and Czepita et al. (2007).^32^

## CONCLUSION

To the best of our knowledge, this is the first research focused on RE of Saudi adult population to evaluate the current state of health service and to plan properly according to our results for better control of the problem in Northern Border Region. The vital epidemiological data on prevalence and patterns of RE from this study are important for planning and improvement of screening and rehabilitation programs for better outcomes of the cases of refractive errors in Arar.
